# Ferroptosis Contributes to Isoflurane Neurotoxicity

**DOI:** 10.3389/fnmol.2018.00486

**Published:** 2019-01-09

**Authors:** Yimeng Xia, Xiaoyun Sun, Yan Luo, Creed M. Stary

**Affiliations:** ^1^Department of Anesthesiology, Ruijin Hospital, Shanghai Jiaotong University School of Medicine, Shanghai, China; ^2^Department of Anesthesiology, Perioperative and Pain Medicine, Stanford University School of Medicine, Stanford, CA, United States

**Keywords:** glutathione peroxidase, ferrostatin-1, iron, reactive oxygen species, mitochondria, volatile anesthetic

## Abstract

The underlying mechanisms of isoflurane neurotoxicity in the developing brain remain unclear. Ferroptosis is a recently characterized form of programmed cell death distinct from apoptosis or autophagy, characterized by iron-dependent reactive oxygen species (ROS) generation secondary to failure of glutathione-dependent antioxidant defenses. The results of the present study are the first to demonstrate *in vitro* that ferroptosis is a central mechanism contributing to isoflurane neurotoxicity. We observed in embryonic mouse primary cortical neuronal cultures (day-*in-vitro* 7) that 6 h of 2% isoflurane exposure was associated with decreased transcription and protein expression of the lipid repair enzyme glutathione peroxidase 4. In parallel, isoflurane exposure resulted in increased ROS generation, disruption in mitochondrial membrane potential, and cell death. These effects were significantly attenuated by pre-treatment with the selective ferroptosis inhibitor ferrostatin-1 (Fer-1). Collectively, these observations provide a novel mechanism for isoflurane-induced injury in the developing brain and suggest that pre-treatment with Fer-1 may be a potential clinical intervention for neuroprotection.

## Introduction

Isoflurane remains a commonly used volatile anesthetic worldwide despite emerging evidence of neurotoxicity to the developing brain (Andropoulos, 2018). Extensive work in rodents has attributed neonatal cognitive dysfunction following inhalational anesthetics exposure to apoptosis, neuroinflammation, reactive oxygen species (ROS) accumulation, neurotransmitter disturbances, and/or changes in synaptic plasticity (Patel and Sun, 2009; Brambrink et al., 2012; Lei et al., 2012; Xia et al., 2017). Mechanistically, isoflurane neurotoxicity has been linked with RhoA-mediated actin depolymerization of the neuronal cytoskeletal architecture (Lemkuil et al., 2011), however inhibition of this process is not clinically protective (Schilling et al., 2017). Therefore, a definitive characterization of the central, underlying mechanisms that regulate neonatal anesthetic toxicity remain a critical knowledge gap impeding the development of interventional therapies.

**Figure 1:**
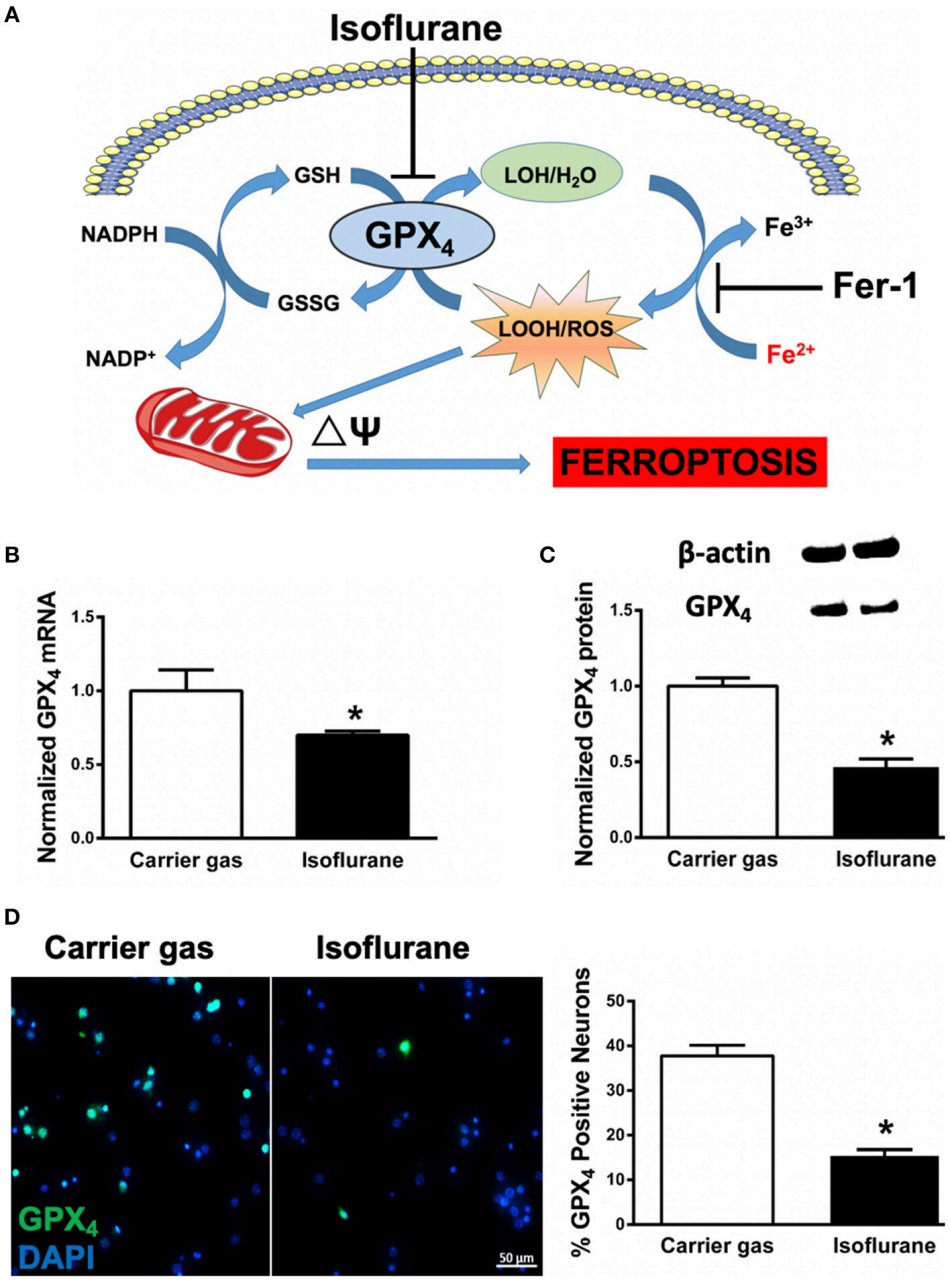
**(A)** Proposed model illustrating ferroptosis in isoflurane neurotoxicity. Exposure of developing neurons to isoflurane suppresses glutathione peroxidase 4 (GPX_4_) expression, leading to augmentation of ferrous- (Fe^2+^) driven formation of reactive lipid peroxides (LOOH) and ROS generation, impairment in mitochondrial membrane potential (ΔΨ) and ferroptosis (cell death). Inhibition by co-administration of ferrostatin-1 (Fer-1) inhibits ferrous-driven formation of LOOH. **(B)** Expression of GPX_4_ mRNA in primary neuronal cultures after isoflurane exposure, normalized to carrier gas exposure. **(C)** Quantification of levels of GPX_4_ protein assessed by immunoblot in neuronal cultures after isoflurane or carrier gas exposure, with example of immunoblot above. **(D)** Immunofluorescent images (left) of GPX_4_ (green) in primary neuronal cultures co-stained with the cell-permeant nuclear dye DAPI (blue). Quantitation of GPX_4_ positive cells (right) as a percentage of total nuclei. All graphs represent *n* = 12 pooled data from three independent experiments. Data are expressed as mean ± SEM. *p < 0.01 compared with the control group. Scale bar = 50 μm. DAPI = 4′,6-diamidino-2-phenylindole, dihydrochloride. Fe^3+^, ferric (iron) ion; GSSG, glutathione disulfide; GSH, glutathione; LOH, non-reactive lipid; NADH^+^, nicotinamide adenine dinucleotide; NADPH, reduced nicotinamide adenine dinucleotide; ROS, reactive oxygen species.

Ferroptosis (Figure 1A), first described in Dixon et al. (2012), is a newly recognized mechanism of programmed cell death characterized by iron-dependent accumulation of lipid peroxides (Angeli et al., 2017; Stockwell et al., 2017; Zilka et al., 2017), driven by inactivation of glutathione peroxidase 4 (GPX_4_, Yang et al., 2014). Emerging evidence suggests ferroptosis contributes to neurodegenerative diseases and brain injury associated with oxidative stress (Skouta et al., 2014), including brain hemorrhage (Li et al., 2017), Alzheimer's disease (Fine et al., 2012; Guo et al., 2013), Huntington's disease (Yang et al., 2005; Niatsetskaya et al., 2010; Speer et al., 2013), stroke (Tuo et al., 2017) and traumatic brain injury (Anthonymuthu et al., 2018). Oxidative stress is associated with neonatal neuronal cell death after inhalational anesthetic exposure (Zhang et al., 2012), however whether ferroptosis contributes to this process has not been investigated. In the present study we assessed GPX_4_ expression, mitochondrial function and cell death in primary neonatal neuronal cultures following isoflurane exposure, with and without pre-treatment with the selective ferroptosis inhibitor ferrostatin-1 (**Fer-1**, Kabiraj et al., 2015).

## Materials and Methods

### Animals and Primary Neuronal Cultures

All animal experiments were approved by Stanford University Animal Care and Use Committee (Stanford, CA, USA) and conducted according to the National Institutes of Health guidelines for animal welfare. Primary cortical neuronal cultures were prepared from embryonic/gestational day 15 or 16 Swiss Webster mice as previously described (Stary et al., 2015). Please refer to Supplementary Material for detailed methods.

### Experimental Protocol

Primary neuronal cultures were pre-treated at day-*in-vitro* (DIV) 7 with 1μM Fer-1 (CAT#SML0583, Sigma Chemicals) a selective ferroptosis inhibitor (Kabiraj et al., 2015; Wu et al., 2018), or 1:5,000 DMSO (Sigma Chemicals) as vehicle control 1 h prior to 6 h of 2% isoflurane exposure or carrier gas (5%CO_2_, 21%O_2_, balance N_2_). Immediately after isoflurane or carrier gas exposure cultures were processed for either reverse transcription–quantitative real-time-PCR, immunoblot, immunocytochemical staining, or live-cell fluorescent imaging of ROS/mitochondrial membrane potential. Please refer to Supplementary Material for detailed methods.

### Reverse-Quantitative Polymerase Chain Reaction (RT–qPCR)

Total RNA was extracted and reverse transcription and PCR were performed as previously described (Ouyang et al., 2012a,b). Ct-values for GPX_4_ were normalized to GAPDH as the internal control and comparisons calculated as the inverse log of the ΔΔCT (Livak and Schmittgen, 2001). Please refer to Supplementary Material for detailed methods.

### Immunoblot

Please refer to Supplementary Material for detailed methods. After protein gel electrophoresis and transfer membranes were blocked and incubated at 4°C overnight with primary antibodies to GPX_4_ (1:500, #125066; Abcam) and anti-β-actin (1:20,000, #A1978; Sigma). Membranes were then incubated with 1:3,000 goat anti-rabbit (CST, #7074) for GPX_4_ and horse anti-mouse (CST, #7076) for β-actin. GPX_4_ band intensity was normalized to β-actin and the isoflurane group then normalized to the carrier gas group.

### Immunocytochemistry

Please refer to Supplementary Material for detailed methods. After fixation cultures were incubated with rabbit monoclonal antibody to GPX_4_ (1:500; ab125066, Abcam) and secondary Alexa Fluor 488-conjugated donkey anti-rabbit (CAT#A-21206, 1:1,000; ThermoFisher Scientific). Cells were visualized at 400X on an inverted Zeiss Observer microscope (Carl Zeiss, Göttingen, Germany).

### Cell Death Assay

Subsequent to isoflurane or carrier gas exposure, cultures were incubated with Hoechst 33342 (5 μM, Sigma) and propidium iodide (PI, 5 μM, Sigma). Automated fluorescent image capture was performed at 200X using a Lumascope^TM^ 720 (Etaluma, Carlsbad, CA). The number of PI-positive and Hoechst-positive cells were quantified using Image J software (v1.49b, National Institutes of Health, USA) and expressed as percentage of total cells.

### Assessment of Reactive Oxygen Species (ROS) and Mitochondrial Membrane Potential

Cultures were incubated with the ROS sensitive dye CellROX^TM^ green (5 μM; #C10444, Life Technologies, Carlsbad, CA) or the mitochondrial membrane potential dye tetramethylrhodamine ethyl ester (TMRE, 50 nM, ThermoFisher Scientific) according to the manufacturer's instructions. Fluorescence was assessed at 200X with a Lumascope^TM^ 720 and fluorescence intensity was quantified using Image J software (v1.49b).

### Statistics

All results are expressed as mean ± standard error (SE). Statistical analysis was performed using SPSS 18.0 software. GPX_4_ mRNA and protein expression levels, CellROS and TMRE fluorescent values were normalized to those of the control group (carrier gas, vehicle alone). All data represent pooled data from 3 individual experiments containing *n* = 4 samples for each treatment group. After normality and equal variance tests, statistical differences between two groups were compared using Student's *t*-test. For data with non-normal distributions, Kruskal–Wallis test was used. For all measurements *p* < 0.05 (95% confidence interval) were considered significant. Please refer to Supplementary Material for detailed methods.

## Results

### Isoflurane Decreases GPX_4_ Expression

**Figure 2:**
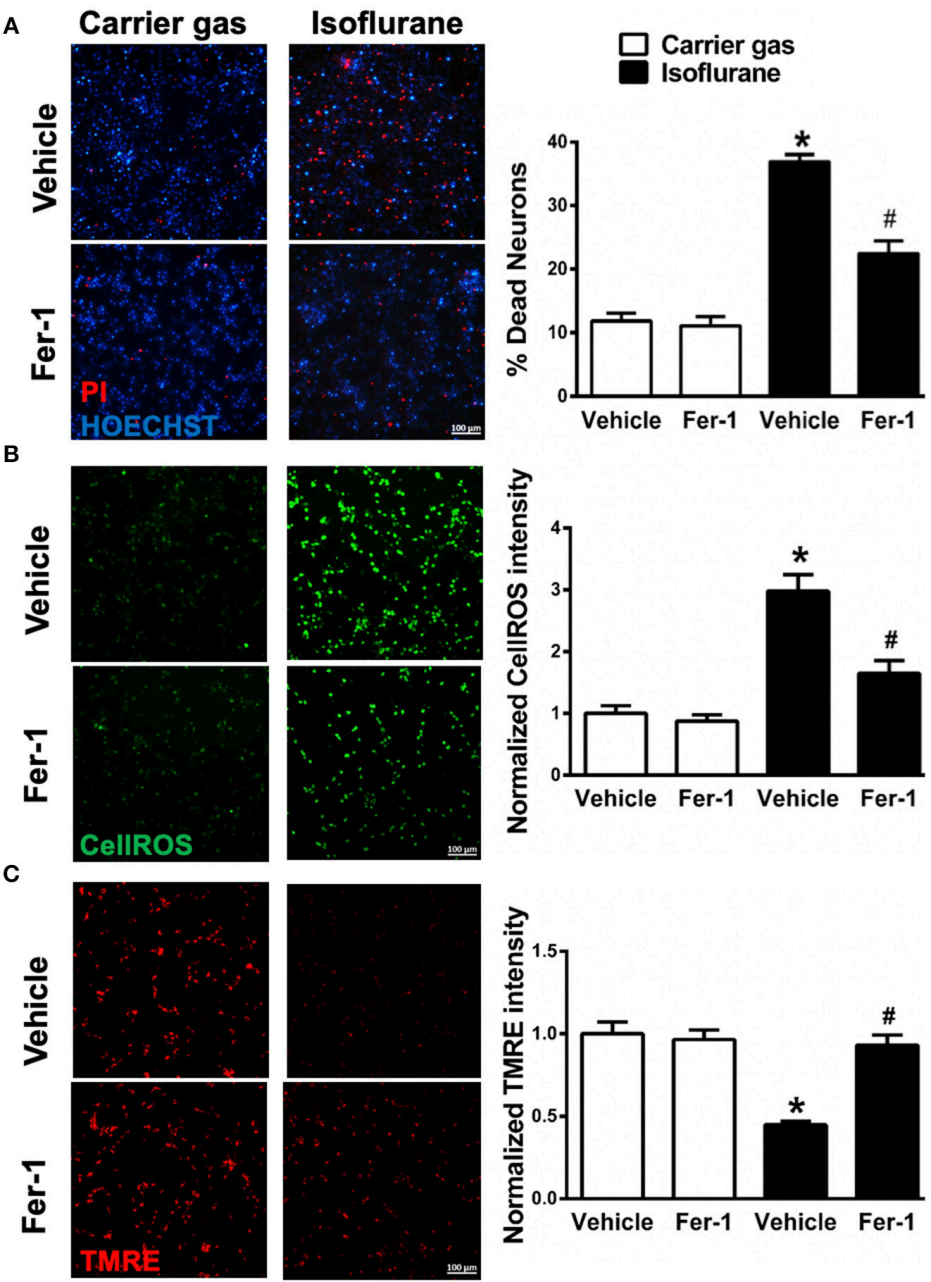
Effects on ferrostatin-1 (Fer-1) on neuronal cell death and mitochondrial function. **(A)** Representative micrographs (left) and quantification of cell death (right) in primary neuronal cultures exposed to isoflurane or carrier gas, with or without Fer-1 pre-treatment, stained with propidium iodide (dead cells, red) and Hoescht (live cells, blue). **(B)** Representative micrographs (left) of ROS generation (CellROX fluorescence, green) and quantitation of fluorescence intensity (right) in primary neuronal cultures exposed to isoflurane or carrier gas, with or without Fer-1 pre-treatment. **(C)** Representative micrographs of mitochondrial membrane potential (TMRE fluorescence, red) and quantitation of fluorescence intensity (right) in primary neuronal cultures exposed to isoflurane or carrier gas, with or without Fer-1 pre-treatment. Graphs represent *n* = 12, pooled data from three independent experiments. All data were expressed as mean ± SEM. *p < 0.01 compared with carrier gas control group; ^#^*p* < 0.01 compared with isoflurane. Scale bar = 100 μm. PI, propidium iodide; ROS, reactive oxygen species; TMRE, tetramethylrhodamine ethyl ester.

In order to assess whether ferroptosis contributes to isoflurane neurotoxicity, we assessed mRNA and protein levels of the central regulator of ferroptosis, GPX_4_ (Dixon et al., 2012; Cardoso et al., 2017). Compared to carrier gas control, GPX_4_ mRNA levels assessed by RT-qPCR significantly (*p* = 0.0035, Student's *t*-test) decreased after 6 h isoflurane treatment (Figure 2B). The mRNA levels of the internal control GAPDH were not statistically different between the two groups. Protein levels of GPX_4_ as assessed by immunoblot were concomitantly significantly (*p* < 0.01, Student's *t*-test) decreased secondary to isoflurane exposure (Figure 2C). In parallel we confirmed a significant (*p* < 0.01, Student's *t*-test) decrease in cellular expression of GPX_4_ by immunocytochemistry (Figure 2D) after isoflurane relative to carrier gas control levels. Together these results demonstrate that 6 h of 2% isoflurane exposure decreases expression of GPX_4_ in these primary neuronal cell cultures.

### Fer-1 Alleviates Isoflurane-Induced Cell Death

To investigate whether ferroptosis contributes to isoflurane neurotoxicity, cell death was assessed in neuronal cultures secondary to isoflurane exposure with and without pre-treatment with the ferroptosis selective inhibitor Fer-1. Isoflurane exposure induced a significant (*p* < 0.01, Kruskal–Wallis test) increase in cell death relative to carrier gas control (Figure 2A), assessed by automated fluorescence microscopy after double staining with Hoescht/PI. Notably, pre-treatment with Fer-1 provided substantial protection by significantly (*p* < 0.01, Kruskal–Wallis test) alleviating cell death by ~50%. Application of Fer-1 had no measurable toxicity in carrier gas control treated cultures (Figure 2A). These findings suggest that ferroptosis contributes to isoflurane neurotoxicity and that Fer-1 pre-treatment is protective in these neuronal cultures.

### Fer-1 Attenuates Isoflurane-Induced ROS Generation

Next, in order to assess whether Fer-1 provides neuroprotection against isoflurane exposure by mitigation of ROS generation CellROX^TM^ green was utilized for live-cell fluorescent imaging. CellROX^TM^ fluorescence increased significantly (*p* < 0.01, Kruskal–Wallis test) in neuronal cultures after isoflurane exposure compared with carrier gas control treatment (Figure 2B). Fer-1 pre-treatment significantly (*p* < 0.01, Kruskal–Wallis test) attenuated CellROX^TM^ fluorescence in isoflurane treated cultures (Figure 2B) but had no effect on CellROX^TM^ fluorescence in carrier gas control treated cultures. These results demonstrate that Fer-1 pre-treatment attenuates ROS generation secondary to isoflurane exposure in these neuronal cultures, likely contributing to observations of improved cell survival.

### Fer-1 Mitigates Isoflurane-Induced Mitochondrial Dysfunction

Finally, we assessed the role of ferroptosis in mitochondrial dysfunction secondary to isoflurane exposure by evaluating mitochondrial membrane potential with and without Fer-1 pre-treatment. Compared with carrier gas control treated cultures, TMRE fluorescence intensity was significantly (*p* < 0.01, Kruskal–Wallis test) depressed following isoflurane exposure (Figure 2C) relative to carrier gas in vehicle-control cultures. However, in Fer-1 treated cultures mitochondrial membrane potential remained largely preserved after isoflurane exposure (Figure 2C). No significant difference in TMRE fluorescence intensity was measured in carrier gas control treated cultures with Fer-1 application. These results demonstrate that Fer-1 mitigates mitochondrial dysfunction secondary to isoflurane exposure.

## Discussion

Proposed mechanisms whereby volatile anesthetics induce cognitive dysfunction during neurodevelopment include GABA-A receptor-mediated Cl^−^ ion efflux and depolarization, disruption in cell-survival signaling pathways leading to neuronal apoptosis, impaired neuronal differentiation, neurogenesis and neuronal migration, and disruption in synaptogenesis and synaptic pruning (for review, see Patel and Sun, 2009; Brambrink et al., 2012; Lei et al., 2012). *In vitro* models utilizing primary neuronal cell cultures have demonstrated that isoflurane only contributes to injury at DIV 4–7 cultures, and not at DIV 10, which roughly parallels the neonatal period *in vivo* brain characterized by active synaptogenesis. Prior studies have demonstrated that mitochondrial function is central to several critical processes for proper brain development (Arrázola et al., 2018; Devine and Kittler, 2018), including neurogenesis, neuronal differentiation, neuronal migration, synaptogenesis and synaptic pruning secondary to programmed apoptosis. Therefore, mitochondria represent a unifying target for therapies aimed at protecting or rescuing the neonatal brain following anesthetic exposure. In the present study, we utilized an isoflurane exposure protocol (2% isoflurane for 6 h) known to induce ROS generation, impair mitochondrial membrane potential and induce cell death in DIV 7 primary neuronal cell cultures (Xie et al., 2006, 2007; Zhang et al., 2010, 2012) in order to assess the potential role of ferroptosis in anesthetic neurotoxicity. We report here for the first time that pre-treatment with the selective ferroptosis inhibitor Fer-1 preserved mitochondrial function and mitigated neuronal cell death secondary to isoflurane exposure (Figure 2), suggesting that ferroptosis represents a central mechanism of anesthetic neurotoxicity in these primary neuronal cell cultures.

Ferroptosis is defined by iron-dependent accumulation of lipid peroxides, and is genetically and biochemically distinct from other forms of programmed cell death such as apoptosis, necrosis and autophagy (Yang and Stockwell, 2016). GPX_4_ is a lipid repair enzyme, which reduces lipid hydroperoxides to lipid alcohols and limit the initiation of ferroptosis (Cao and Dixon, 2016; Tonnus and Linkermann, 2016). Inhibition of GPX_4_ activity leads to rapid accumulation of lipid peroxides and cell death, and deletion of GPX_4_ is embryonic lethal (Seiler et al., 2008; Yang et al., 2014), therefore GPX_4_ expression has been used as a marker for ferroptosis (Cardoso et al., 2017; Conrad et al., 2018). In the present study we observed a significant decrease in GPX_4_ mRNA and GPX_4_ protein expression with isoflurane exposure (Figure 1), supporting our hypothesis that isoflurane induces ferroptosis. These observations, in combination with our parallel observations that mitochondrial dysfunction secondary to isoflurane exposure were largely reversed by pre-treatment with Fer-1 (Figure 2), imply a central role for ferroptosis in isoflurane neurotoxicity. Future *in vivo* studies selectively altering GPX_4_ expression are needed to more accurately assess the mechanistic contribution of decreased GPX_4_ to isoflurane neurotoxicity.

Whether general anesthetics have the capacity to permanently impair cognitive function remains controversial (Hudson and Hemmings, 2011; Zhou et al., 2015), and clinical trials in matched cohorts testing protective compounds may provide the only avenue for direct evidence of neurotoxicity in children. In the present study we observed that Fer-1 had no effect on ROS generation, mitochondrial membrane potential or cell death in neuronal cultures, suggesting that Fer-1 may provide a clinically safe therapeutic intervention. However, one limitation of the present study is that cell cultures were grown at atmospheric partial pressure of O_2_ (21%), which represents a hyperoxic state relative to physiological O_2_ levels in the brain which approach 1–8% (Erecinska and Silver, 2001). Our group have previously described differences in mitochondrial function between cultures grown at 21% and cultures grown at 5% O_2_ (Sun et al., 2015), suggesting that *in vitro* experiments assessing mitochondrial mechanisms should account for this. Second, although 2% isoflurane *in vitro* treatment represents *in vivo* physiologic levels of exposure (Herold et al., 2017), a 6 h duration of single agent exposure would not represent a typical pediatric anesthetic. Further studies extending these findings to clinically relevant *in vivo* models utilizing multiple anesthetic agents and more common exposure durations are required to advance Fer-1 as a potential neuroprotectant in pediatric patients requiring anesthesia.

## Author Contributions

YX planned and executed experiments and drafted the manuscript. XS assisted with experiments. YL assisted in intellectual design and reviewed manuscript. CS assisted in methodological planning, experimental execution, data review, and manuscript preparation.

### Conflict of Interest Statement

The authors declare that the research was conducted in the absence of any commercial or financial relationships that could be construed as a potential conflict of interest.
