# Supplementary material for: Ferroptosis Contributes to Isoflurane Neurotoxicity
Source: Front Mol Neurosci. 2019 Jan 9;11:486. doi: 10.3389/fnmol.2018.00486 (PMC6333734; doi:10.3389/fnmol.2018.00486)
Supplement: Supplementary file 1 [file Data_Sheet_1.PDF]

DETAILED MATERIALS AND METHODS

Animals and primary neuronal cultures

All animal experiments were approved by Stanford University Animal Care and Use Committee (Stanford, CA, USA) and conducted according to the National Institutes of Health guidelines for animal welfare. Primary cortical neuronal cultures were prepared from embryonic/gestational day 15 or 16 Swiss Webster mice as previously described (Stary et al., 2015). In brief, embryonic cortices were collected in ice-cold Eagle's minimal essential medium, digested with 0.05% trypsin/EDTA for 15 min at 37°C, mechanically dissociated, then plated in Dulbecco's modified Eagle's medium containing 26 mM $NaHCO_3$ (Sigma Chemicals, St.Louis, MO), 24 mM glucose (Sigma Chemicals), 5% fetal bovine serum and 5% equine serum.

Experimental protocol

Primary neuronal cultures were pre-treated at day-in-vitro (DIV) 7 with 1μM Fer-1 (CAT#SML0583, Sigma Chemicals) a selective ferroptosis inhibitor (Kabiraj et al., 2015; Wu et al., 2018), or 1:5000 DMSO (Sigma Chemicals) as vehicle control 1h prior to 6h of 2% isoflurane exposure or carrier gas (5%CO_2, 21%O_2, balance N_2). Gas and anesthetic concentrations were monitored using a Datex 245 Airway Gas Monitor (Datex Corp., North Clearwater, Fl, USA) and gas flow was maintained at 2 L/min in a sealed and humidified incubator at 37°C. Immediately after isoflurane or carrier gas exposure cultures were processed for either reverse transcription–quantitative real-time-PCR (RT-qPCR), immunoblot, immunocytochemical staining, or live-cell fluorescent imaging of ROS/mitochondrial membrane potential.

Reverse-quantitative polymerase chain reaction (RT–qPCR)

Total RNA was extracted as previously described (Ouyang et al., 2012a; Ouyang et al., 2012b)

with TRIzol® (ThermoFisher Scientific, Waltham, MA). Reverse transcription was performed with TaqMan mRNA Reverse Transcription Kit (ThermoFisher Scientific) as previously described (Ouyang et al., 2012a; Ouyang et al., 2012b). PCR was performed as previously described (Ouyang et al., 2012a; Ouyang et al., 2012b) using predesigned primers for GPX_4 (#Mm00515041,ThermoFisher Scientific) and glyceraldehyde-3-phosphate dehydrogenase (GAPDH, #Mm99999915,ThermoFisher Scientific). Ct values for GPX_4 were normalized to GAPDH as the internal control and comparisons calculated as the inverse log of the ΔΔCT (Livak and Schmittgen, 2001). Molecular assays were prepared in duplicate, and experiments were independently performed in triplicate.

Immunoblot

Following isoflurane exposure, cultures were immediately lysed in ice-cold RIPA buffer with protease and phosphatase inhibitors. BCA assay was utilized to assess protein concentration and 100 μg of protein/sample was separated on a 4–10% Bis-Tris mini-gel (NP0304BOX, ThermoFisher Scientific), and electro-transferred to Immobilon polyvinylidene fluoride membrane (IPVH00010, Millipore EMD Corp.). Membranes were blocked in 5% nonfat milk and incubated at 4°C overnight with primary antibodies to GPX_4 (1:500 dilution, catalog no.125066; Abcam) and anti-β-actin (1:20000 dilution, catalog no. A1978; Sigma). Membranes were then washed and incubated with 1:3,000 goat anti-rabbit (CST, #7074) for GPX_4 and horse anti-mouse (CST, #7076) for β-actin, and immunoreactive bands were visualized using a chemiluminescent imaging system (Azure C300, Azure Biosystems, CA). Densitometric analysis was performed using Image J software (vl.46, National Institutes of Health) by an observer blinded to treatment group. GPX_4 band intensity was normalized to β-actin and the isoflurane group then normalized to the carrier gas group.

Immunocytochemistry

Cultures were rinsed three times with phosphate-buffered saline (PBS) and treated with 4% paraformaldehyde in PBS for 45 min, followed by treatment with blocking buffer (5% horse serum and 0.1% Triton PBS) for 1h at room temperature. Cultures were incubated with rabbit monoclonal antibody to GPX_4 (1:500; ab125066, Abcam, Cambridge, United Kindom) diluted in blocking buffer at 4 °C overnight followed by incubation with secondary Alexa Fluor 488-conjugated donkey anti-rabbit (CAT#A-21206, 1:1000; ThermoFisher Scientific). Cells were visualized at 400X using a 40x Zeiss LD Plan Neofluar air objective on an inverted Zeiss Observer microscope (Carl Zeiss, Göttingen, Germany). The microscope was equipped with for epifluorescence with LED-based excitation at 488nm with Zeiss filter set #38 (excitation bandpass 470/40nm, beam splitter 495nm, emission bandpass 525/50nm) and controlled with Zen (v2 Blue Edition, Carl Zeiss) software.

Cell death assay

Subsequent to isoflurane or carrier gas exposure, cultures were incubated with Hoechst 33342 (5 µM, Sigma Chemicals) and propidium iodide (PI, 5 µM, Sigma Chemicals). PI penetrates cells with compromised cell membranes to stain nuclei red while Hoechst freely penetrates to label both live and dead cell nuclei blue. Automated fluorescent image capture was performed at 200X (20X Olympus LUCPlan LFN air objective, 3 fields per well) using a Lumascope™ 720 (Etaluma, Carlsbad, CA) to eliminate observer/selection bias. The appropriate Lumascope™ filter sets include: excitation 370-410nm, emission 429-462nm for Hoechst; and excitation 580-598nm, emission 612-680 for PI. The number of PI-positive and Hoechst-positive cells were quantified by an observer blinded to treatment group using Image J software (Image J, v1.49b, National Institutes of Health, USA) and expressed as percentage of total cells as previously described (Stary et al., 2015).

Assessment of reactive oxygen species (ROS) and mitochondrial membrane potential

Immediately after isoflurane or carrier gas exposure cultures were incubated with the ROS sensitive dye CellROX^{TM} green (5 μM final concentration; C10444, Life Technologies, Carlsbad, CA) according to the manufacturers' instructions. The cell-permeant probe is weakly fluorescent in a reduced state and exhibits bright green fluorescence upon oxidation by ROS and subsequent binding to DNA. To assess mitochondrial membrane potential cells were incubated with tetramethylrhodamine ethyl ester (TMRE, 50 nM, ThermoFisher Scientific) for 30 min according to the manufacturer's instructions immediately prior to isoflurane exposure. CellROX green and TMRE red fluorescence were assessed using automated fluorescent image capture (200X, 3 fields per well with the Lumascope^{TM} 720, at excitation intensities that neither induced photobleaching or phototoxicity. The relevant Lumascope^{TM} filter sets include: excitation 473-491nm, emission 505-561nm for CellROX^{TM} green; and excitation 580-598nm, emission 612-680 for TMRE. Fluorescence intensity was quantified by an observer blinded to conditions using Image J software (v1.49b).

Statistics

All results are expressed as mean ± standard error (SE). Statistical analysis was performed using SPSS 18.0 software. GPX_4 mRNA and protein expression levels, CellROS and TMRE fluorescent values were normalized to those of the control group (carrier gas, vehicle alone). All data represent pooled data from 3 individual experiments containing n=4 samples for each treatment group. For RT-qPCR and immunoblots, a single data point represents cell material from 4 individual wells combined. Each experiment consists of n=4 samples for each treatment group, so graphs represent n=12 samples (or 48 wells) per treatment group. For cell imaging experiments, each data point represents 4 wells, with 5-6 pictures taken for each well and averaged as a single data point. Graphs therefore represent n=12 samples for each treatment from the three combined dissections (or 48 wells total with 5-6 images each). After normality and equal variance tests, statistical differences between two groups were compared using Student's

t-test. For data with non-normal distributions, Kruskal-Wallis test was applied for statistical comparison. For all measurements $p<0.05$ (95% confidence interval) were considered statistically significant.
